# Astrocytic but not Microglial Antigen Presentation Shapes Protective Immunity to Toxoplasma gondii in the Brain

**DOI:** 10.21203/rs.3.rs-9544619/v1

**Published:** 2026-05-11

**Authors:** Sydney A. Labuzan, Anne E. Schuster, Michael A. Kovacs, Maureen N. Cowan, Mark J. Lawson, Isaac W. Babcock, Lydia A. Sibley, Abigail G. Kelly, Anne E. Marchildon, Tajie H. Harris

**Affiliations:** University of Virginia; University of Virginia; University of Virginia; University of Virginia; University of Virginia; University of Virginia; University of Virginia; University of Virginia; University of Virginia; University of Virginia

**Keywords:** Antigen Presentation, Microglia, Astrocytes, Neuroimmunology, Toxoplasma gondii

## Abstract

T cells play a pivotal role in orchestrating immune defense within the central nervous system (CNS) during many infections. *Toxoplasma gondii*, a brain-trophic protozoan parasite, establishes lifelong CNS infection that remains largely subclinical in immunocompetent hosts but can cause severe encephalitis in immunocompromised individuals. While CD8^+^ T cells are essential for controlling *T. gondii* during chronic infection through both cytokine production and cytolytic killing, the CNS-resident cells that functionally present antigen in the brain to promote T cell function or serve as cytolytic targets remain incompletely defined. Here, we investigated the contributions of CNS-resident macrophages and astrocytes, two key CNS-resident cell types, antigen presentation during chronic *T. gondii* infection. Using mice lacking MHCI or MHCII in CNS-resident macrophages, we found no impairment of immune responses or ability of the brain to control parasite, indicating dispensable function of resident macrophages as APCs during infection. However, deletion of MHCI on astrocytes led to deficits in parasite control, in turn promoting elevated CD4^+^ T cell cytokine production and recruitment of iNOS^+^ inflammatory monocytes. We observed increased presence of lytic parasite within the brain, which suggests that astrocyte MHCI may be necessary to control parasite replication throughout the CNS. Our findings underscore a previously underappreciated role for astrocytic MHCI within the CNS during infection and highlights the dispensability of CNS-resident macrophages to this process.

## BACKGROUND

T cells emerge as critical orchestrators of the immune response during many central nervous system (CNS) infections, shaping host-protective mechanisms essential for host survival^1,2^. One prominent brain-tropic pathogen is *Toxoplasma gondii*, an intracellular protozoan parasite that establishes a lifelong chronic infection in the CNS across a range of mammalian hosts, including humans^3,4^. In immunocompetent individuals, *T. gondii* infection is typically asymptomatic due to effective immune control. However, in immunocompromised populations, such as individuals with AIDS or organ transplant recipients, reactivation of the parasite can lead to toxoplasmic encephalitis (TE), a potentially fatal uncontrolled infection of the brain^5^. In murine models, deficiencies in T cell responses lead to unchecked parasite proliferation and lethal TE, underscoring the indispensable role of T cells in controlling chronic *T. gondii* infection^1,3,6–14^.

Although the CNS is no longer considered strictly immune-privileged, it retains unique immunological features^15–17^. Under homeostatic conditions, the brain exhibits minimal expression of antigen-presenting machinery and lacks conventional antigen-presenting cells (APCs), such as dendritic cells^15,16^. In the setting of a neurotropic pathogen, like *T. gondii*, this prompts a central question of how CD8^+^ T cell responses are sustained and how infected cells are recognized and eliminated within the CNS. Studies have shown that microglia, the resident innate immune cells of the brain, upregulate antigen presentation machinery following an insult to the brain, including in the context infection or neurodegeneration^18–26^. This suggests one mechanism by which the brain becomes a more immunologically reactive tissue. The blood-brain barrier (BBB) at steady-state serves as a stringent gatekeeper to limit immune cell infiltration into the brain parenchyma^27^. During infection with *T. gondii*, the brain vasculature becomes activated to allow the robust recruitment of parasite-specific T cells into the brain parenchyma^28–30^. Of note, once recruited, T cells can only persist within the tissue if their cognate antigen is expressed at this site^31,32^.

There is growing interest in understanding the dual role of CD8^+^ T cells in the brain, where they can act as both pathogenic and protective agents during infection, neurodegeneration, and injury^20,27,33–39^. It is known that coordinated interferon-gamma (IFNγ) production by both CD4^+^ and CD8^+^ T cells is a critical component of the immune response to *T. gondii*^1,7,9,14,40–42^. In addition to cytokine production, CD8^+^ T cells mediate host-protection through cytolytic killing, recognizing infected cells via Major histocompatibility complex I (MHCI) antigen presentation, and eliminating them through perforin- and granzyme-dependent mechanisms^1,41,43–45^. Perforin production by CD8^+^ T cells was found to be dispensable in the peripheral phase of *T. gondii* infection, but necessary for control of cyst burden in the chronic brain phase of infection^13^. This demonstrates that cytolytic killing by CD8^+^ T cells is an integral component of immunity to *T. gondii* in the brain. Conventional type 1 dendritic cells (cDC1s) have been shown to be a key antigen-presenting cell type that promotes expansion of parasite-specific T cells at peripheral sites during early *T. gondii* infection^1,7,46,47^. However, these cells largely remain at the brain borders during infection and are rarely found within deeper brain parenchyma, leaving it an open question as to which cells interact with CD8^+^ T cells once they transit into the brain^48^.

CNS antigen presentation to date has been most thoroughly studied in experimental autoimmune encephalitis (EAE) where antigen-specific T cells infiltrate the CNS parenchyma and drive disease pathogenesis^49,50^. In this context, dendritic cells recruited into the CNS parenchyma have been identified as critical APCs via Major histocompatibility complex class II (MHCII) while microglial presentation proved dispensable^51,52^. Recent literature has expanded our understanding of antigen presentation within the CNS, revealing unexpected complexity among resident and infiltrating cell types. Microglia have been shown to upregulate MHCI and II molecules during infection and to putatively cross-present antigen to CD8^+^ T cells in the context of vesicular stomatitis virus^18,20,36^. Despite rarely found actively infected by *T. gondii*, this cross-presentation would allow microglia to display phagocytosed antigen via MHCI or II to stimulate T cell function or be targeted for cytolytic killing^3,25,53^. Several studies have implicated microglia, perivascular macrophages, and brain endothelial cells (BECs) in priming CD8^+^ T cells for CNS entry during viral infections^20,25,34,54^. Although microglia are widely recognized as professional APCs in the CNS, the potential for other resident cells to present antigen for targeted cytolytic killing remains unknown.

Astrocytes are glial cells well known for their homeostatic functions in maintaining the BBB, metabolizing glutamate, stabilizing extracellular concentration of potassium, and producing trophic survival factors for neurons and other glia^55^. Foundational studies, largely *in vitro*, recognized astrocytes as non-traditional APCs that upregulate antigen presentation machinery in autoimmune disorders and infection^55–61^. In the context of *T. gondii*, astrocytes play a critical role in parasite containment through IFN-driven responses and form heterogeneous reactive subpopulations during chronic infection^62–64^. Neurons are the predominant cell type with active infection of *T. gondii*, with a smaller subset of astrocytes observed harboring parasite throughout the course of infection^53^. As such, neurons and astrocytes represent potential sources of *T. gondii* antigen for presentation via MHCI to CD8^+^ T cells, with the capacity to either promote T cell function, act as targets of cytolytic killing, or contribute to both processes. Using a mouse model in which neurons express the H2-L^d^ allele, an MHCI variant known to drive an immunodominant, protective CD8^+^ T cell response, studies have implicated neuronal antigen presentation in controlling parasite burden during *T. gondii* infection^65^. Neurons have also been shown, along with microglia, to contribute to the shaping of resident memory T cell populations in a model of latent *T. gondii* infection^24,65^. These studies point to potential roles of antigen presentation by infected cells during chronic neuroinflammatory states. Despite these recent advances, significant gaps remain in our understanding of how CNS-resident cells use antigen presentation to shape CD8^+^ T cell function and control of parasite during chronic neuroinflammation.

In this study, we aimed to define how CNS-resident macrophage and astrocyte antigen presentation modulates T cell responses and parasite control during chronic *T. gondii* infection. To address this question, we employed cell type-specific knockouts of the *B2m* gene, a critical component of the MHCI complex, on astrocytes and CNS-resident macrophages. We hypothesized that ablation of MHCI expression in one or both cell types would impair CD8^+^ T cell–mediated immunity or targeted killing, leading to increased parasite burden in the brain. We additionally generated a CNS-specific macrophage knockout of MHCII to examine how this would impact CD4^+^ T cell function and parasite burden in the brain. MHCI or MHCII-deficient CNS-resident macrophages displayed no immune impairment, suggesting dispensable function or compensation by other cells presenting antigen within the CNS. We found that loss of MHCI in astrocytes resulted in impaired parasite control during chronic infection. We also observed heightened immune activity, including elevated cytokine production by infiltrating CD4^+^ T cells and increased production of iNOS^+^ by inflammatory monocytes. These findings reveal a unique role for astrocyte antigen presentation in parasite control during chronic CNS infection and provide new insight into the specialized immunological landscape of the brain.

## RESULTS

### Microglia upregulate antigen presentation machinery during chronic Toxoplasma gondii infection.

**Figure 1:**
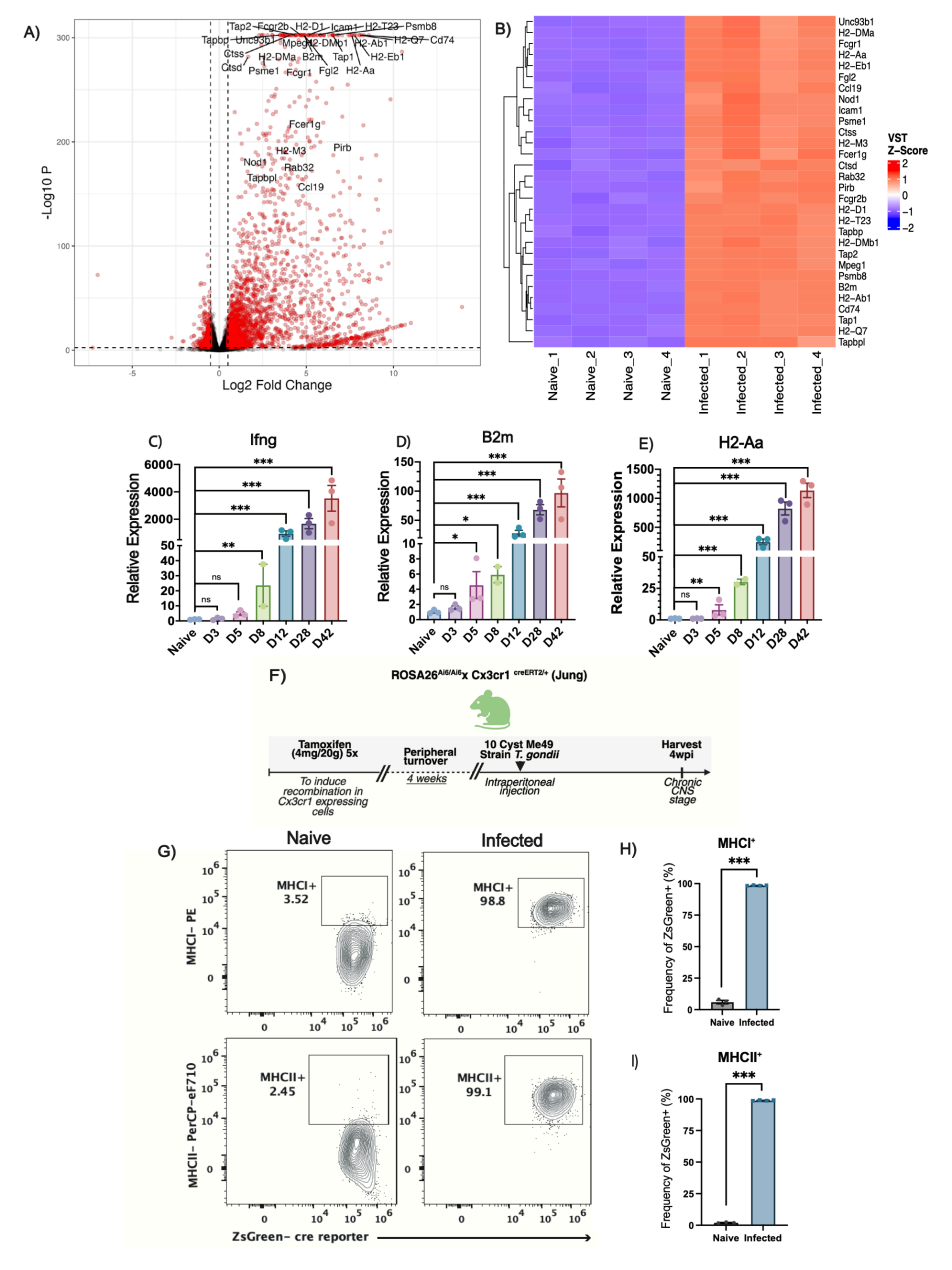
Antigen presentation machinery is upregulated in the CNS in response to *T. gondii* infection. C57BL/6 mice were mock-infected with PBS or infected intraperitoneally (i.p.) with 10 cysts of the Me49 strain of *T. gondii*, and whole brains were harvested at 4 weeks post-infection (4wpi) for bulk RNA-sequencing. **A)** Volcano plot indicating differential gene expression between whole brain samples for naïve and chronically infected mice, with top 30 DEGs from the GO Term “Antigen processing and presentation” (GO: 0019882) indicated on plot. **B)** Heatmap displaying these top 30 DEGs in naïve and infected brains. **(C-E)** RT-qPCR analysis of Ifng **(C)**, B2m **(D)**, and H2-Aa **(E)** expression in whole mouse brain across harvested at seven timepoints from naïve to 42dpi. Data normalized to naïve timepoint. **F)** Experimental paradigm for labeling of ZsGreen+ CNS-resident macrophages and infection. **G)** Representative flow cytometry gating for CD11b+CD45intZsGreen+ microglia MHCI and MHCII expression in naïve and infected samples. **(H-I)** Flow cytometric quantification of microglial MHCI and MHCII expression at naïve and 4wpi. n=4 mice/group (**A-B**), n=2-3 mice/timepoint (**C-E**), in n=3-4 mice/group (**G-I**). For **A-B** statistical significance was defined in the differential gene expression analysis as an adjusted p value < 0.05. For **C-E** data were log_2_-transformed and evaluated using one-way ANOVA with Tukey’s post hoc multiple comparisons test. For **H-I**, statistical significance determined using unpaired t-test, Data are presented as mean ± s.e.m., *** = p < 0.001. **(F)** made using Biorender.com.

To examine the timing of this upregulation during infection, RT-qPCR analysis of whole brain homogenate from mice harvested at various timepoints from day 0 (naive) to day 42 (D42) post-infection was performed. Interferon-γ (IFNγ) promotes the upregulation of antigen presentation machinery, including MHC molecules, in brain-resident cells during inflammation^18,21,36,66^. When we examined dynamics of gene expression of *Ifng* across infection, we observed an increased pattern of expression from 8 dpi up until 42 dpi (Fig. 1C). The gene *β2m*, a key component of the MHCI complex, followed a similar pattern of expression across infection in the brain (Fig. 1D). Further, gene expression of *H2-Aa*, a key MHC Class II gene, by RT-qPCR showed a similar upregulation, with a peak during chronic infection (Fig. 1E). Together, these data indicate that CNS infection is accompanied by a coordinated, sustained induction of IFNγ and both MHCI and MHCII gene expression, peaking during chronic infection. Observing these changes in the whole brain across infection, we aimed to hone in on in which CNS-resident cells’ antigen presentation is functionally relevant for control of infection.

Due to their role as the innate immune cell of the brain, we hypothesized microglia would serve as robust antigen presenters during chronic infection. We used *ROSA26*^Ai6/Ai6^ x *Cx3cr1*^CreERT2+/−^ mice, which express ZsGreen fluorescence specifically in *Cx3Cr1*^+^ cells following cre activity induced by the application of tamoxifen^67^. We administered five intraperitoneal (i.p.) injections of tamoxifen. We then waited four weeks for peripheral monocyte turnover, leaving labeling confined to longer-lived CNS-resident macrophages. We then mock-infected with PBS or infected mice i.p. with 10 cysts of the Type II strain of *T. gondii* Me49 (Fig. 1F). Brains were harvested at 4 weeks post-infection (4 wpi) and flow cytometry was performed to examine antigen presentation markers **(Fig. S1A)**.

Upon infection, we measured robust populations of MHCI and MHCII expressing cells not present in the naïve brain. Preparing brain tissue for flow cytometric analyses results in a single cell suspension predominantly composed of immune cells and lacking CNS-resident cell types. To explore the immune cell populations expressing MHCI and MHCII during infection we categorized individual immune populations. At the whole brain level, concurrent with our RT-qPCR results, we observe very low expression of MHCI in the naïve state, but robust upregulation during infection **(Fig. S1B)**. Within the infected brain, as we expected, infiltrating immune cells abundantly express MHCI (~ 44% Ly6C^hi^ and ~ 19% Ly6C^low^ monocytes), and microglia upregulate MHCI expression (Fig. 1G–H, S1C). Correspondingly, we observe low expression of MHCII in the naïve state, but robust upregulation during infection **(Fig. S1D)**. Within the MHCII^+^ population in the infected brain, we see this population comprised predominantly of infiltrating monocytes (~ 42% Ly6C^hi^ and ~ 25% Ly6C^low^ monocytes) and smaller populations of B cells and microglia (~ 7% B cells and ~ 6% microglia) **(Fig. S1E)**. Small numbers of dendritic cells (cDC1s and cDC2s) expressing both MHCI and MHCII are found within the meninges at this time point but are rarely found in the deeper brain parenchyma^32^.

Narrowing in on the CD45^int^CD11b^+^ZsGreen^+^ microglial population, as expected, we observed minimal expression of MHCI or MHCII molecules in the naïve state (~ 2–3%) but significant upregulation of these molecules during chronic infection in the brain (~ 98–99%), reflecting a similar pattern previously observed as early as 12dpi in the brain (Fig. 1G–I, **Fig. S1A**)^18^. Although microglia comprised a relatively minor fraction of MHCI and MHCII-expressing cells compared to infiltrating immune cells, their drastic shift in expression of antigen presentation machinery highlighted them as candidates for promoting T cell function during CNS infection.

### CNS-resident macrophage MHCI- antigen presentation is dispensable for control of T. gondii during chronic infection.

**Figure 2:**
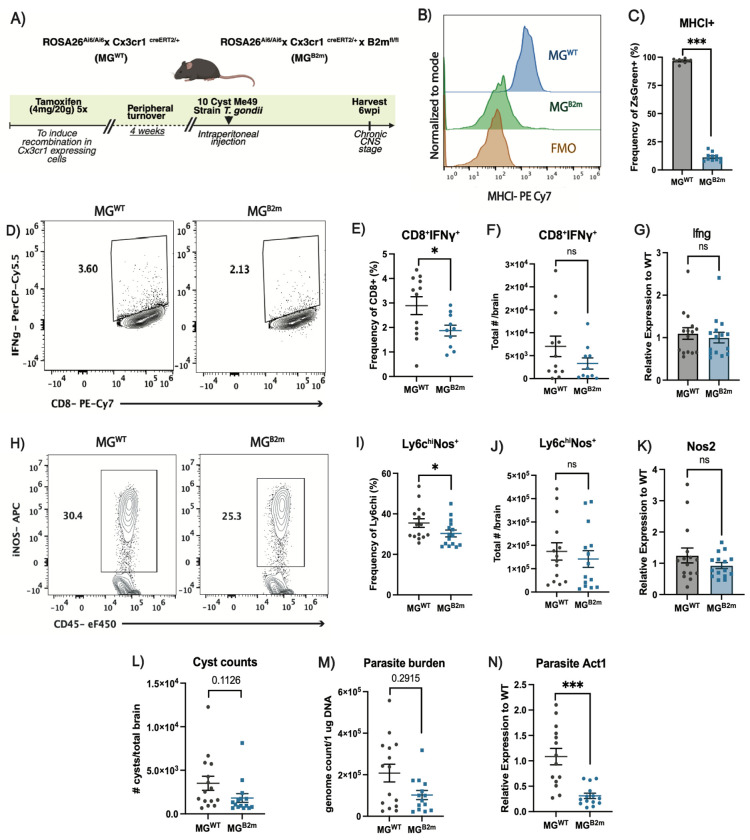
CNS-resident macrophage MHCI is dispensable for control of *T. gondii* infection within the brain. **A)** Experimental schematic for tamoxifen administration and infection of MGWT and MGB2m groups. **B)** Representative histogram of MHCI expression on ZsGreen+ microglia. **C)** Frequency of ZsGreen+ microglia expressing MHCI to examine efficacy of excision in MGB2m compared to MGWT mice. **D)** Representative flow gating for CD8+IFNg+ populations. Flow cytometric quantification of **E)** frequency of IFNg+ within the TCRb+CD8+ population and **F)** total number of TCRb+CD8+IFNg+ cells in the brains in MGWT and MGB2m mice. **G)** RT-qPCR analysis of Ifng in whole mouse brain of MGWT and MGB2m mice. **H)** Representative flow gating for iNOS+ (CD45hiCD11b+Ly6G-Ly6ChiNOS+) populations. Flow cytometric quantification of **I)** frequency and **J)** total number of iNOS+ within the CD45hiCD11b+Ly6G-Ly6Chi population in the brains of MGWT and MGB2m mice. **K)** RT-qPCR analysis of Nos2 in whole brains of MGWT and MGB2m mice. To quantify parasite burden: **L)** manual cyst counts **M)** RT-qPCR for genomic parasite DNA and **N)** RT-qPCR for *T. gondii*-specific Act1. Statistical significance was determined via randomized block ANOVA compiled from 3-4 experiments with n=10-12 mice/group **(C-F),** and n=15 mice/group **(G, I-N).** Data are presented as mean ± s.e.m; * *p* < 0.05, ***p< 0.001, ns; not significant. **(A)** made using Biorender.com.

Since we observe this robust upregulation of MHCI during infection by microglia, we wanted to elucidate the functional relevance of this to promoting T cell function and control of parasite. To address this question, we generated *ROSA26*^Ai6/Ai6^ x *Cx3cr1^CreERT2+/−^ x B2m^fl/fl^* mice, hereafter referred to as MG^B2m^, to ablate MHCI from *Cx3Cr1*^+^ cells and allow fluorescent ZsGreen-labeling of these cells. We induced deletion of the *β2m* gene via five doses of tamoxifen administration. We waited four weeks to make the deletion specific to CNS-resident macrophages (i.e. microglia and border associated macrophages) and infected mice i.p. with 10 cysts of the Me49 *T. gondii* strain. Mice progressed to the chronic phase of infection and brains were analyzed at 6 wpi (Fig. 2A). We examined immune responses in the brain at this timepoint using flow cytometry **(Fig. S2A)**.

We first examined efficacy of the genetic deletion and observed that compared to controls (MG^WT^) (~ 98%), on average only ~ 13% of microglia in the brains of knockout mice (MG^B2m^) expressed MHCI during chronic infection (Fig. 2B–C). Microglia lacking MHCI had no change in sufficiency for expression of MHCII (**Fig. S3A**). By flow cytometry, we observed no differences in the total number of TCRβ^+^ T cells, or TCRβ^+^CD4^+^ and TCRβ^+^CD8^+^ T cells in the brains of knockout mice during chronic infection (**Fig. S3B-D**). Established mechanisms of parasite control in the brain include T cell–derived IFNγ and tumor necrosis factor–α (TNFα), along with downstream inducible nitric oxide synthase (iNOS) production by infiltrating inflammatory monocytes^3^. When assessing functionality of these CD8^+^ T cells to produce cytokine, we plated and incubated cells *ex vivo* with Brefeldin A (BFA). We observed decreased production of IFNγ when mice lack MHCI on CNS-resident macrophages by percentage of the overall CD8^+^ population (Fig. 2D–E) but not by number (Fig. 2F). At the transcriptional level, we observed no difference in *Ifng* levels in the brains of knockout mice compared to WT mice (Fig. 2G). Additionally, we observed no differences in TNFα production by CD8^+^ T cells (**Fig. S3E-F**) or at the whole brain RNA level (**Fig. S3G**). We observed within the CD4^+^ T cell population a decrease in production of both IFNγ and TNFα in our knockout mice by frequency but not by overall number present in the brain (**Fig. S3H-K**). Collectively, these results demonstrate that loss of MHCI in CNS-resident macrophages does not alter T cell effector function, as measured by cytokine production.

As a further measure of immune control, we measured production of iNOS by Ly6C^hi^ infiltrating myeloid cells and found decreased percentage in the knockout mice (Fig. 2H–I). However, this did not correspond to a decrease in overall number of Ly6C^hi^iNOS^+^ infiltrating myeloid cells (Fig. 2J) or any difference in overall *Nos2* RNA level in the brains of these mice (Fig. 2K). These results suggested an overall unimpaired immune response when CNS-resident macrophages lack MHCI, likely due to dispensable function or compensatory antigen presentation by other cells in the brain.

Finally, to assess how microglial antigen presentation affects control of parasite, we used three measures of parasite burden: cyst counts performed by brightfield microscopy, qPCR assay of parasite genomic DNA, and parasite *Act1* gene expression from whole brain samples. While observing no significant differences in the quantity of cysts or abundance of parasitic genomic DNA (Fig. 2L–M), we did observe a decrease in gene expression of parasite *Act1* in the knockout mice (Fig. 2N). Given the lack of increased parasite burden and overall preserved immune responses, this suggests that MHCI by CNS-resident macrophages is dispensable for parasite control during chronic infection.

### Loss of MHC Class II expression in CNS-resident macrophages does not impact immune control of T. gondii in the brain

**Figure 3:**
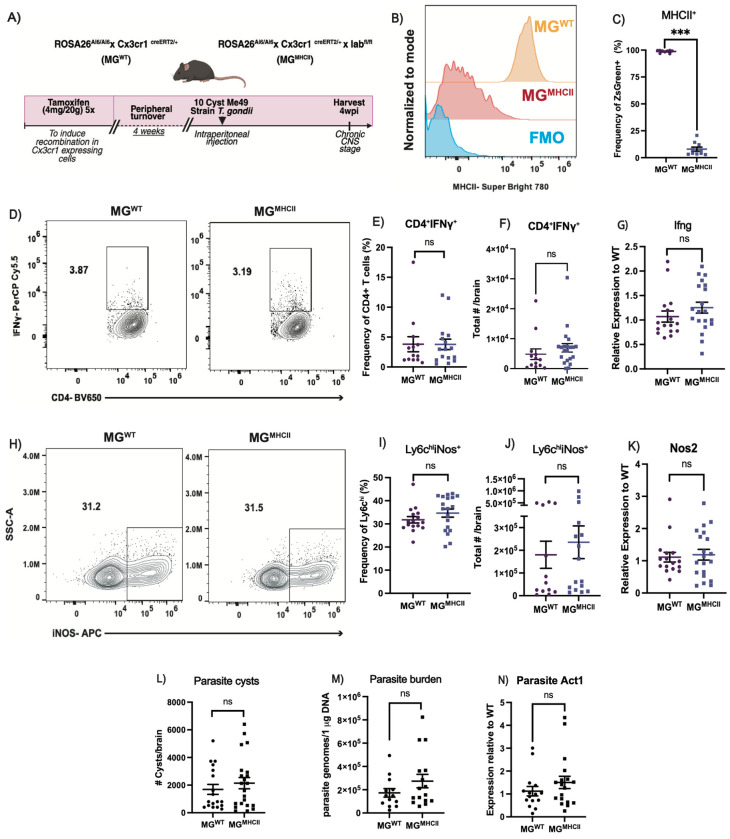
CNS-resident macrophage MHCII deletion does not impact immune responses or control of CNS parasite burden. **A)** Experimental schematic for tamoxifen administration and infection. **B)** Representative histogram of MHCII expression by ZsGreen+ microglia in MGWT and MGMHCII and **C)** frequency of ZsGreen+ microglia expressing MHCII to examine efficacy of knockdown of MHCII in MGMHCII mice compared to MGWT. **D)** Representative flow gating for TCRb+CD4+IFNg+ populations. Flow cytometric quantification of **E)** frequency and **F)** total number of IFNg+ within TCRb+CD4+ cells in the brain. **G)** RT-qPCR analysis of Ifng in whole mouse brain. **H)** Representative flow gating for iNOS+ (CD45hiCD11b+Ly6G-Ly6ChiNOS+) populations. Flow cytometric quantification of **I)** frequency and **J)** total number of iNOS+ within the CD45hiCD11b+Ly6G-Ly6Chi population in the brain. **K)** RT-qPCR analysis of Nos2 in whole mouse brain. To quantify parasite burden: **L)** manual cyst counts **M)** RT-qPCR for genomic parasite DNA and **N)** RT-qPCR for *T. gondii*-specific Act1. Statistical significance was determined via randomized block ANOVA compiled from 3-4 experiments with n=9-10 mice/group **(C),** and n=13-16 mice/group **(E-G, I-N).** Data are presented as mean ± s.e.m; ***p< 0.001, and ns; not significant. **(A)** made using Biorender.com.

In addition to upregulating MHCI, we observed that CNS-resident macrophages robustly upregulate expression of MHCII during chronic *T. gondii* infection (Fig. 1G–I)^18^. Previous work showed that when microglia lack the transcription factor *Stat1* and cannot respond to IFN-signaling, they do not upregulate MHCII and succumb to *T. gondii* infection^18^. Having observed no significant deficit in immune control when CNS-resident macrophages lack MHCI expression, we hypothesized that these cells may preferentially present phagocytosed parasitic antigen via the MHCII pathway. To test this hypothesis, we generated *Rosa26*^Ai6/Ai6^ x *Cx3cr1^CreERT2+/−^ x Iab^fl/fl^* (MG^MHCII^) mice that ablate MHCII from CNS-resident macrophages. We administered tamoxifen at 4–6 weeks of age, waited for 4 weeks, and mice were then subsequently infected. Mice were analyzed at a chronic infection timepoint of 4wpi by flow cytometry (Fig. 3A, **Fig. S2A)**.

We examined efficacy of the genetic deletion and observed that compared to controls (MG^WT^) (~ 98%), on average only ~ 10% of microglia in the brains of knockout mice (MG^MHCII^) expressed MHCII during chronic infection (Fig. 3B–C). We found MG^MHCII^ mice remained sufficient for MHCI expression (**Fig. S4A**). We hypothesized that if CNS-resident macrophage MHCII presentation plays a role in sustaining T cell responses during chronic *T. gondii* infection, a disruption in CNS-resident macrophage MHCII expression would result in reduced effector functions of CD4 T cells. Thus, we analyzed this population first by flow cytometry. By flow cytometry, we observed no differences in the total number of TCRβ^+^ T cells, or TCRβ^+^CD4^+^ and TCRβ^+^CD8^+^ T cells in the brains of knockout mice during chronic infection (**Fig. S4B-D**).

We observed no deficits in production of IFNγ by CD4^+^ T cells either by measures of frequency of total CD4^+^ T cells (Fig. 3D–E) or in number of CD4^+^IFNγ^+^ T cells (Fig. 3F). Additionally, we observed no differences in overall level of *Ifng* in the brains of these mice at the RNA level (Fig. 3G). When assessing production of the cytokine TNFα, we observe an increase in CD4^+^TNFα^+^ T cells by frequency of all CD4^+^ T cells in MG^MHCII^ brains, but no difference in overall number (**Fig. S4E-F**). At the whole brain level, there is also no difference in *Tnf* gene expression between MG^MHCII^ and MG^WT^ mice (**Fig. S4G**). We also observed no change in CD8^+^ T cell IFNγ and TNFα production by flow cytometry when microglia lack MHCII (**Fig. S4H-K**). Taken together, these results demonstrate that loss of MHCII in microglia does not affect T cell effector function, as measured by cytokine production.

We further examined downstream production of iNOS by Ly6C^hi^ infiltrating inflammatory monocytes and observed no changes by percentage of iNOS^+^ monocytes (Fig. 3H–I) or by total number present in the brain (Fig. 3J). Further confirming these results, we found no difference in *Nos2* expression at the RNA level in our microglial knockouts (Fig. 3K). Additionally, we observed no difference in parasite burden by any measures when CNS-resident macrophages lacked MHCII expression (Fig. 3L–N). These results from our CNS-resident macrophage MHCI and MHCII knockouts suggest a dispensable role for microglial antigen presentation in the context of *T. gondii* infection and prompted us to question a role for other CNS-resident cells within the CNS.

### Astrocytes upregulate MHC Class I expression during chronic T. gondii infection.

**Figure 4:**
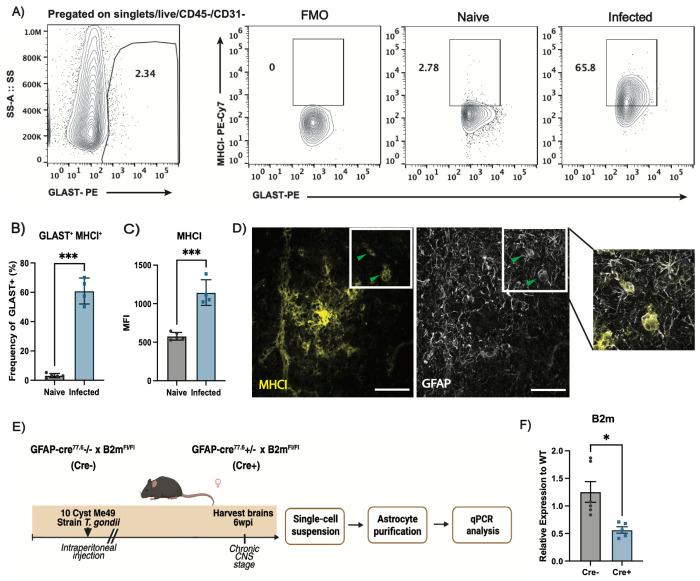
Astrocytes upregulate MHCI in response to *T. gondii* infection. **A)** Representative flow cytometry plots showing MHCI expression on GLAST^+^ astrocytes from naïve and 6wpi infected brains, pre-gated on singlets, live cells, and CD45^−^CD31^−^ populations. **B)** Frequency and **C)** Mean Fluorescence Intensity (MFI) of GLAST^+^ astrocytes expressing MHCI in naïve and infected mice. **D)** Representative IHC images showing MHCI (yellow) expression and GFAP^+^ astrocytes (white) in infected brain tissue. Scale bar= 100 μm. Inset highlight colocalization of MHCI^+^ GFAP+ astrocytes (arrowheads). **E)** Experimental schematic for astrocyte-specific deletion of *B2m* using *GFAP-Cre × B2mfl/fl* mice during chronic infection. **F)** RT-qPCR analysis examining B2m expression in purified ACSA2-astrocytes from Cre^+^ mice compared to Cre^−^ controls. n= 4-5 mice/group **(B-C)** and n= 5-6 mice/group **(F).** Statistical significance determined using unpaired t-test, data presented as mean ± s.e.m. *p < 0.05, ***p < 0.001.

While traditionally appreciated for their roles in maintenance of neuronal health and homeostasis, astrocytes have recently emerged as critical players in neuroimmunity^55,56,62,68,69^. Despite being shown to upregulate antigen presentation machinery and stimulate T cell proliferation *in vitro*, the capacity of astrocytes to act as antigen presenters *in vivo* remains ambiguous^56,60,61^. To investigate whether astrocytes are a relevant cell type in presenting antigen to CD8^+^ T cells during *T. gondii* infection, C57BL/6 mice were either mock-infected with PBS or infected with 10 cysts of the Type II strain Me49 and brains harvested 6 weeks later to assess expression of astrocytic MHCI. Through flow cytometry analysis, we found GLAST+ cells had very little expression of MHCI (~ 2%) in the PBS-injected naïve group, compared to a significant increase in MHCI during infection (~ 60%) (p < 0.001) (Fig. 4A–B, **Fig. S5A)**. Further, by Mean Fluorescence Intensity (MFI) we observe a significant increase in the abundance of MHCI present on the GLAST^+^ population during infection (Fig. 4C). Additionally, in brains stained by immunofluorescence from chronically infected mice, we observe colocalization of GFAP^+^ cells with MHCI staining, further indicative of upregulation of MHCI by astrocytes (Fig. 4D).

To determine how astrocytic MHCI expression dictates CD8^+^ T cell function in the brain during infection, we crossed *Gfap-77.6*- cre to *B2m*^fl/fl^ mice to constitutively excise the gene *B2m* from astrocytes. *Gfap77.6*-cre^−/−^-*B2m*^fl/fl^ (Cre−) control and *Gfap77.6*-cre^+/−^-*B2m*^fl/fl^ (Cre+) mice were infected with 10 cysts of the type II Me49 strain of *T. gondii* and brains harvested 6 weeks later (Fig. 4E). To confirm excision efficiency of the *B2m* gene in the knockout mice, ACSA2^+^ astrocytes were purified, and RT-qPCR was performed for the gene *B2m*. We observed an approximate ~ 50% reduction in *B2m* gene expression in *Gfap77.6*-cre^+/−^-*B2m*^fl/fl^ mice (Cre+) compared to control *Gfap77.6*-cre^−/−^-*B2m*^fl/fl^ (Cre−) mice (Fig. 4F). To examine intact capacity to present by more traditional APC populations, CD45^int^CD11b^+^ CNS-resident macrophages and infiltrating CD45^hi^CD11b^+^ macrophages were assessed by flow cytometry (**Fig. S5B**). We found canonical APCs were unaffected in *Gfap*-cre^+/−^*B2m*^fl/fl^ (Cre+) mice and were sufficient for MHCI expression comparable to controls (Cre−) mice (**Fig. S5C-F**).

### Mice deficient in astrocytic MHC class I display impaired parasitic control during chronic infection.

**Figure 5:**
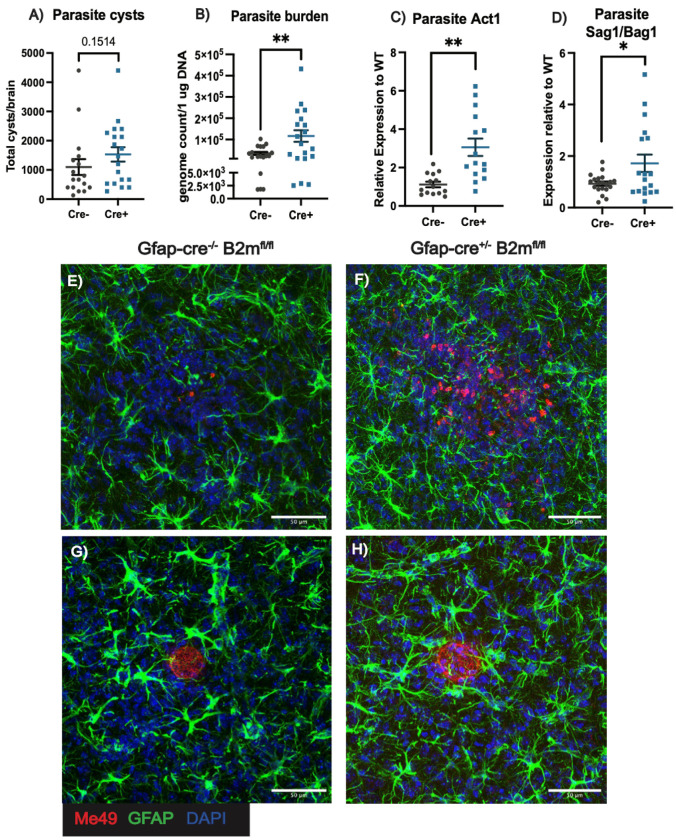
Astrocyte-specific MHCI deletion increases *T. gondii* parasite burden in the brain. **(A)** Total counts of parasite cysts per brain in *GFAP-Cre−/− B2mfl/fl* (Cre−) and *GFAP-Cre+/− B2mfl/fl* (Cre+) mice. **(B)** qPCR of *T. gondii* genomic DNA isolated from the brain. **(C)** RT-qPCR of parasite-specific **Act1 from whole brain RNA**. **(D)** Ratio of parasite **Sag1 (tachyzoite-specific gene) to Bag1** (bradyzoite-specific gene) normalized to Act1 expression. **(E–H)** Representative immunofluorescence images of infected brain tissue from *GFAP-Cre^−^/−* (**E,G**) and *GFAP-Cre+/−* (**F,H)** *B2m*fl/fl mice showing parasites (Me49, red), astrocytes (GFAP, green), and nuclei (DAPI, blue). Scale bar = 50 μm. **(A-D)** Statistical significance was determined via randomized block ANOVA compiled from 4 experiments with n=16-19 mice/group. Data are presented as mean ± s.e.m; *p < 0.05, **p < 0.01.

To first assess how knockdown in astrocytic MHCI antigen presentation would affect control of parasite, we used three measures of parasite burden. We observed no difference in cyst counts between knockout (Cre+) and wildtype (Cre−) mice (Fig. 5A). However, by total parasite genomic DNA and *Act1* gene expression, we observed increased parasite burden in the brains of mice with deletion of MHCI from astrocytes (Fig. 5B–C). Further, we performed qPCR of *Sag1* and *Bag1*, genes specific to tachyzoite and bradyzoite parasite stages, respectively. We observe an increased ratio of *Sag1/Bag1* present when mice lack astrocytic MHCI, indicating an increase in the tachyzoite form of the parasite (Fig. 5D). This suggests an impairment of control of parasite replication. To visualize parasite in the brains of these mice, immunofluorescence with an astrocytic marker (GFAP) and an antibody against the Me49 parasite was performed. In agreement with our quantitative parasite burden data, we observed notable areas of tachyzoites (Fig. 5E–F) but no difference in the cyst form of the parasite (Fig. 5G–H).

### Astrocyte MHC Class I-deficiency leads to increased CD4 + T cell cytokine production.

**Figure 6:**
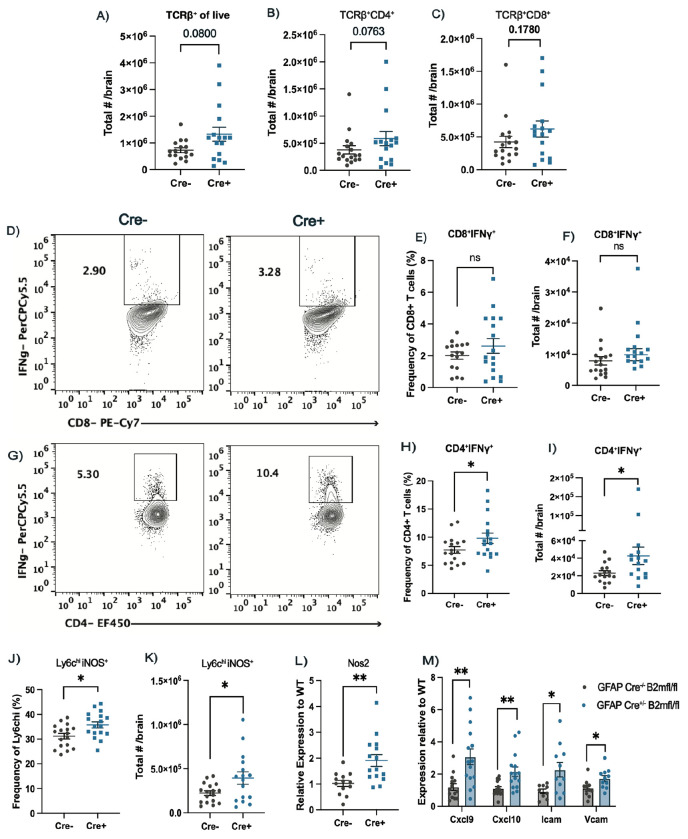
Astrocyte MHCI deletion enhances CD4^+^ T cell–driven immune responses. **A)** Total numbers of TCRβ^+^ T cells, **B)** CD4^+^ TCRβ^+^ T cells, and **C)** CD8^+^ TCRβ^+^ T cells in the brains of *GFAP-Cre−/−* and *GFAP-Cre*+/− *B2mfl/fl* mice at 6wpi. **D)** Representative flow plots and quantification of the **E)** frequency and **F)** total number of CD8^+^IFNγ^+^ T cells. **G)** Representative flow plots and quantification of the **H)** frequency and **I)** total number of IFNγ^+^ CD4^+^ T cells. **J)** Frequency and **K)** total number of iNOS^+^ Ly6Chi inflammatory monocytes in the brain. **L)** RT-qPCR analysis of Nos2 expression in whole brain homogenate. **M)** RT-qPCR expression of chemokine and adhesion molecule genes (**Cxcl9, Cxcl10, Icam1, Vcam1**) in whole brain tissue. Statistical significance was determined via randomized block ANOVA compiled from 2-4 experiments with n=16-19 mice/group (**A-C, E-F, H-I, J-L**), n=9-14 mice/group (**M**) data are represented as mean ± s.e.m. *p < 0.05, **p < 0.01; ns, not significant.

Observing the increase in parasite burden in knockout mice, we next assessed CD8^+^ and CD4^+^ T cell function when mice lack astrocytic MHCI. We hypothesized if astrocyte MHCI expression played a key role in coordinating CD8^+^ T cell function, we would observe decreased effector T cell functions, such as cytokine (IFNγ and TNFα) and Granzyme B production. To assess their function, flow cytometric analysis was performed for immune cell populations at the 6-week timepoint. We observed no overall differences in the number of overall TCRß^+^ T cells (Fig. 6A), TCRß^+^CD4^+^ T cells (Fig. 6B), and TCRß^+^CD8^+^ T cells in the brains of knockout mice (Fig. 6C). When we assessed production of the key cytokine IFNγ, we observed no deficits in production by TCRß^+^CD8^+^ T cells either by frequency (Fig. 6D–E) or by overall number (Fig. 6F). We also observed no differences in the production of TNFɑ by TCRß^+^CD8^+^ T cells when mice lack astrocytic MHCI (**Fig. S6A-B**). Intriguingly, we did observe increases in TCRß^+^CD4^+^ T cells producing IFNγ by frequency and number (Fig. 6G–I), as well as those producing the cytokine TNFα (**Fig. S6C-D)**.

To confirm astrocyte antigen presentation does not impact the capacity of T cells to produce cytokine, brain cells were stimulated *ex vivo* with PMA/ionomycin. We observed no differences in TCRß^+^CD8^+^ capacity to produce IFNγ and TNFα (**Fig. S6E-F).** PMA/ionomycin stimulated TCRß^+^CD4^+^ cells from brains lacking astrocytic MHCI did possess higher capacity to produce IFNγ but not TNFα when compared to controls (**Fig. S6G-H).** To examine deficits in cytotoxic function by CD8^+^ T cells when astrocytes lack MHCI, staining was additionally performed for Granzyme B (GrzmB). We observed no difference in the production of Granzyme B by frequency, number, or MFI of TCRß^+^CD8^+^ T cells (**Fig. S6I-K**). Further, at the whole brain RNA level, we observed no change in the amount of *GrzmB* transcript in the Cre+ animals (**Fig. S6L**). Overall, these results suggest that there is not a deficit in cytotoxic potential or cytokine production by CD8^+^ T cells. These results suggest that CD8^+^ T cell function is not directly affected, whereas altered CD4^+^ T cell responses point to increased inflammation as an indirect consequence of astrocyte MHCI deletion, due to increased parasite burden.

Since we observed increases in cytokine production by CD4^+^ T cells, we hypothesized we would observe downstream effects on IFNγ-dependent processes, including the production of anti-parasitic iNOS by infiltrating monocytes. We observed no change in the total number of infiltrating myeloid cells when mice lack astrocytic MHCI **(Fig. S7A)**. To assess production of iNOS by these cells, we performed flow cytometric analysis and observed increases in both the frequency of iNOS^+^Ly6c^hi^ inflammatory monocytes (Fig. 6J), as well as total number in the brains of mice lacking astrocytic MHCI (Fig. 6K). Further, we observed increased level of *Nos2* gene expression in the brain when mice lack astrocyte MHCI (Fig. 6L). Further, we found a decreased frequency in CD4^+^Foxp3^+^ regulatory T cells within the total CD4^+^ T cell population, but ultimately similar number of regulatory T cells between groups (**Fig. S7C-D**). This increased proportion of CD4^+^ effector T cells supports findings of an overall enhanced inflammatory response to the increased parasite in the knockout mice. We further found increased expression in IFN-driven genes, including chemoattractants *Cxcl9* and *Cxcl10* and adhesion molecules *Icam* and *Vcam*, which correspond with overall enhanced inflammation in the brains of the knockout mice (Fig. 6M).

When assessing the spleen as an indicator of systemic immune activation in Cre+ knockout mice, we found no differences in the overall numbers of TCRß^+^ T cells, or TCRß^+^CD4^+^ and TCRß^+^CD8^+^ subsets (**Fig. S7E-F**). We observed no difference in the numbers of proliferative CD4^+^ and CD8^+^ T cells within the spleens of these mice compared to controls at this timepoint (**Fig. S7F**). Taken together, this suggests that the enhanced immune response is specific to the brains of these knockout animals. These observations indicate that when astrocytes lack the ability to present antigen to cytotoxic T cells, parasite replication increases, and leads to an enhanced immune response driven by cytokine producing CD4^+^ T cells.

## DISCUSSION

Our study reveals that MHCI antigen presentation by astrocytes, but not CNS-resident macrophages, is important in control of *Toxoplasma gondii* replication within the brain. We found that when CNS-resident macrophages lacked MHCI or MHCII expression, we observed no deficits in parasite control or immune responses. However, we demonstrate that the absence of MHCI expression on astrocytes results in an increase in parasite. The increase in parasite led to enhanced rather than deficient immune responses driven by CD4^+^ T cell cytokine production and downstream monocyte iNOS production. These findings highlight a previously undescribed role for how astrocytes present antigen to promote control of *T. gondii* in the CNS. These results provide not only greater insight into host–parasite interactions in the brain, but also how T cell recognition of infected astrocytes plays a role in control of pathogen.

Much of the work in the neuroimmune space has emphasized microglia as the central APCs and modulators of neuroinflammatory responses within the CNS^19–21,25,26,36,51,56,70–74^. More recently, this perspective has expanded, as interest grows in how T cells contribute to brain immunity across diverse contexts, from infectious diseases to neurodegenerative disorders. This shift reflects a broader recognition that antigen presentation in the CNS is more complex and multifaceted than previously thought.

Our group previously demonstrated that interferon signaling in microglia is essential for control of *T. gondii*, as mice lacking *Stat1* in microglia succumb early in chronic infection^18^. Interferon signaling drives robust upregulation of antiparasitic effector pathways, including *Irg* and *Gbp* family genes, along with antigen presentation machinery, highlighting the importance of interferon in enabling microglia to mount an effective response^18^. Despite rarely being found infected *in vivo* in mice, when microglia lack *Stat1* signaling, microglia were found to be harboring parasite, suggesting an innate ability to clear parasite by these cells^18^. While these findings establish microglia as indispensable for parasite control, the results of the present study indicate that antigen presentation itself is a dispensable component of this protective role.

Findings across disease models illustrate the context-dependent nature of microglial antigen presentation in the CNS. In EAE, dendritic cells rather than microglia appear to be the dominant APCs coordinating CD4^+^ T cell responses, consistent with our observation that microglial MHCII is dispensable during chronic *T. gondii* infection^51,52^. Moreover, our data align with previous findings demonstrating that loss of *Tap* in microglia does not affect total T cell populations or cytokine production in the brains of mice during latent *T. gondii* infection^24^. In peripheral tissues, infected cDC1s are shown to play a key role in presentation of *T. gondii* to promote an immune response, however they largely remain at the brain borders during chronic infection^47,48,75^. Our data demonstrate that CD11b^+^CD45^hi^Ly6C^low^ and Ly6C^hi^ infiltrating peripheral populations constitute the largest contributors to the overall pool of MHCI^+^ and MHCII^+^ cells in the brain during infection. Thus, these cells could be poised to act as the predominant APCs throughout *T. gondii* infection, compensating for any microglial dysfunction.

By contrast, in a viral model of Theiler’s murine encephalomyelitis virus (TMEV), microglia and perivascular macrophages act as APCs to promote CD8^+^ T cell infiltration into the brain^20^. Similarly, studies in tauopathy have shown that microglia regulate T cell entry and function in ways that exacerbate disease pathology^76^. A key distinction between these models is the degree of peripheral myeloid cell infiltration into the brain. Some viral infections and neurodegenerative models exhibit limited recruitment of peripheral APCs to the brain parenchyma, whereas chronic *T. gondii* infection and EAE involve substantial infiltration of monocytes and dendritic cells, respectively. Together, these findings suggest that microglia may play a more prominent APC role in settings where infiltrating professional APCs are scarce, while in highly inflammatory contexts with abundant peripheral APCs their contribution may be comparatively dispensable.

Neurons, as the principal cell observed with active infection, would be expected to encounter the highest levels of parasite-derived antigen. It is now appreciated that MHCI expression by neurons during development is crucial for synaptic pruning and refinement, but whether neurons upregulate and use MHCI functionally during disease remains unclear^77,78^. Recent studies have aimed to understand how MHCI expression by neurons may play a role in control of *T. gondii*. *In vitro* primary murine neurons respond to IFNγ to upregulate *Irg*s, *Gbp2*, *Stat1*, and *Mhc1*, as well as pretreatment with IFNγ leads to decreased levels of *T. gondii* infected neurons^79^. In a latent infection model of *T. gondii* where C57BL/6 mice were generated to possess a floxed MHCI immunoprotective *H2-L^d^* allele, knocking out *H2-L^d^* from neurons results in greater cerebral parasite burden^65^. These findings raise additional questions about how this process occurs *in vivo* in C57BL/6 mice, which express the *H2-D* and *H2-K* MHCI alleles, and whether neurons that present antigen can directly interact with cytotoxic CD8^+^ T cells.

Although neurons represent the predominant cell type harboring *T. gondii* in the brain, a small subset of infected astrocytes has been identified through the use of a Cre-secreting parasite^53^. In this system, *T. gondii* is engineered to secrete Cre-recombinase into host cells, enabling fluorescence in cells injected with parasite effector proteins during invasion^53^. The relative rarity of infected astrocytes despite widespread parasite exposure suggests that these cells possess intrinsic or extrinsic immune-mediated mechanisms for parasite clearance, a topic that has received increasing attention in the field. Previous work has shown the necessity of *Stat1*-mediated interferon-signaling in astrocytes in restricting *T. gondii* burden in the brain^80^. This finding was inferred to be a deficit in the ability to upregulate anti-parasitic machinery, thus allowing astrocytes to serve as a residential niche for parasite^80^. Recently, it was shown that astrocytes do not use caspase 8-mediated apoptosis as a parasite restriction mechanism during *T. gondii* infection^81^. While *in vitro* studies have observed upregulation of MHCI by astrocytes and interactions in co-cultures with CD8^+^ T cells, to date it has not been thoroughly explored how antigen presentation by astrocytes may contribute to host defense from pathogens *in vivo* within the CNS^60,61,66,82,83^. Our findings directly address this gap by demonstrating that astrocytic MHCI upregulation is one mechanism for pathogen restriction during CNS infection. We propose that this effect is mediated through interactions with cytotoxic CD8^+^ T cells, enabling detection of and elimination of infected astrocytes.

## CONCLUSION

Together, our findings establish astrocytes as a functionally important source of antigen presentation required for effective control of *T. gondii* in the CNS. Although microglia robustly upregulate antigen presentation machinery during infection, their antigen presentation is dispensable for parasite control. By contrast, astrocyte MHCI expression plays a role in limiting pathogen burden, likely through display of parasite antigen by infected cells. This work provides novel insight into host–pathogen dynamics within the infected brain, where professional immune cells such as microglia, while highly reactive, do not appear to function as critical APCs in promoting T cell function. Instead, effective immune control appears to depend in part on antigen presentation by infected parenchymal cells themselves. Given their abundance and association with neurons and sites of parasite reactivation, astrocytes are positioned to serve as sites of CD8^+^ T cell immune surveillance and targeted pathogen clearance. However, this has not been thoroughly explored *in vivo*, a gap addressed in the present study. Our finding gives new insight into host-pathogen dynamics and likely extends to other disease contexts in which astrocytes are directly infected, underscoring their broader role as sources of antigen that drive pathogen control through MHCI upregulation.

## METHODS

### Animals and Treatments:

*Gfap ^Cre77.6^* (#024098), *Cx3cr1^CreERT2^* (#020940), *Iab1*^fl/fl^ (#037709), ROSA26^Ai6/Ai6^ (#007906), and CBA/J (#000656) strains were obtained from the Jackson Laboratory and maintained within UVA’s animal facility. Swiss Webster (#024) mice were purchased from Charles River Laboratories. *B2m^fl/fl^* mice were generously provided by Dr. Wayne Yokoyama from Washington University. Cre lines were bred with *B2m^fl/fl^* mice to produce *Gfap*^cre/+^ x *B2m^fl/fl^*, *ROSA26* ^Ai6/Ai6^ x *Cx3cr1^CreERT+/−2^ x B2m^fl/fl^*, and *ROSA26* ^Ai6/Ai6^ x *Cx3cr1^CreERT2+/−^ x H2-Ab1^fl/fl^* mouse lines.

The Me49 type II strain of *T. gondii* was maintained *in vivo* and passaged through chronically infected (3–12 months) Swiss Webster and CBA/J mice. For experimental infections with the Me49 strain, tissue cysts were prepared from homogenized brains of chronically infected (3–8 weeks) CBA/J mice. Mice were then inoculated i.p. with 10 tissue cysts of Me49 in 200 μl of 1X PBS (Gibco Cat#14190144). Mice infected and used for studies were monitored and euthanized if they showed weight loss greater than 20% of their pre-infection bodyweight.

### Tamoxifen treatment:

To induce cre-expression and excision of *B2m* and *Iab1* for the Cre^ERT2^ driven mouse lines (*Cx3cr1^CreERT2^)*, tamoxifen (Sigma-Aldrich Cat#T5645) was dissolved in corn oil (Sigma-Aldrich Cat#C8267) and filtered through a 0.45 μm filter (Millipore Cat#SLGSM33SS). At four to six weeks old, age and sex-matched mice were i.p. injected with tamoxifen (200 mg/kg) every other day for a total of five injections. Four weeks was allowed for turnover of peripheral macrophages prior to parasite infection.

### RNA sequencing analysis:

RNA reads from FASTQ files were trimmed and filtered using Trimmomatic (v0.39) paired end set to phred 33 quality scoring. Adapters were trimmed, and reads with a minimum quality score of 15, leading and trailing quality scores of 3, and minimum fragment length of 36 were used for analysis. FastQC (v0.11.9) was used to verify quality of sample reads. Trimmed and filtered reads were aligned to thee GENCODE M13 reference genome using Salmon (v0.8.2) and output as sam files. Transcript abundance files were imported into R (v4.1.1) and converted to gene abundances using Tximport (v1.24.0). The R Bioconductor package, DESeq2 (v1.36.0), was used to perform differential expression analysis. DESeq2-normalized data was visualized using the following R packages: ComplexHeatmap (v2.25.2) and ggplot2 (v4.0.2). Gene names were converted from mouse ENSEMBL gene identifiers to gene symbols using the Bioconductor BiomaRT (v2.52.0) database. Labeled genes were manually selected from significantly differentially expressed genes from the DESeq2 results data frame. All genes with a Benjamini-Hochberg (BH) adjusted p-value below 0.05 were considered significantly upregulated if they had a log2FC > 0.5, and downregulated if they had a log2FC < −0.5. Enrichment score is reported as the −log10 of enrichment p value, based on Kolmogorov-Smirnov (KS) analysis. For targeted analysis of antigen presentation and processing genes, the GO term was used, and the top 30 expressed genes were plotted.

### Parasite burden quantification:

DNA was isolated from whole brain homogenate using the Isolate II Genomic DNA Kit (Bioline, BIO-52067). Prior to isolation, brains were first homogenized in 1X PBS using the Omni TH tissue homogenizer (Omni International). Amplification of *T. gondii* 529 bp repeat region using the SensiFAST Probe No-Rox Kit (Bioline, BIO-86005) and CFX384 Real-Time System (Bio-Rad) was performed as previously described^84^. Tissue DNA (500 ng) was loaded into each reaction. *T. gondii* isolated from human foreskin fibroblasts (HFFs) was used to generate a serial standard curve from 3-300,000 genome copies and determine the number of *T. gondii* genomes per μg of tissue DNA. To measure brain parasite by cyst counts, whole brains were first placed in 4 mL of complete RMPI and passed through an 18-gauge and then 23-gauge (BD, Cat# 305155) needle to homogenize tissue. 30 μL of brain homogenate was then mounted on a slide and *T. gondii* cysts were manually counted using a DM2000 LED bright-field microscope.

### RT-qPCR:

Brain homogenate was inoculated in Trizol (Fisher Scientific Cat#15-596-026). RNA was extracted according to manufacturer’s (Invitrogen) protocol. cDNA was then generated using a High-Capacity Reverse Transcription Kit (Applied Biosystems Cat# 4374967). Quantitative PCR was performed using 2X Taq based Master Mix (Bioline Cat#21105) and Taq Man gene expression assays (ThermoScientific Cat#4331182). Samples were run on a CFX384 Real-Time System thermocycler (Bio-Rad Laboratories). Genes were normalized to murine *Hprt* and the 2^(−ΔΔCT)^ method was used to analyze relative expression^85^. The following Thermofisher mouse gene probes were used: *Hprt* (Mm00446968_m1), *Ifng* (Mm01168134_m1), *B2m* (Mm00437762_m1), *H2-Aa* (Mm00439211_m1), *Grzmb* (Mm00442837_m1), *Tnf* (Mm00443258_m1), *Nos2* (Mm00440502_m1), *Cxcl9* (Mm00434946_m1), *Cxcl10* (Mm00445235_m1), *Icam* (Mm00516023_m1), *Vcam* (Mm01320970_m1). Custom primers for used for analyzing *T. gondii* genomic DNA and gene expression were used as previously described^18^.

### Tissue processing for flow cytometry:

After transcardiac perfusion of mice using 20 mL of cold 1X PBS, brains were collected into cold complete RPMI media (cRPMI; 10% FBS [Gibco], 1% penicillin/streptomycin [Gibco], 1% sodium pyruvate [Gibco], 1% non-essential amino acids [Gibco], and 0.1% 2-Mercaptoethanol [Life Technologies]). Brains were then passed through an 18-gauge and 23-gauge needle for mechanical homogenization. For immune cell isolation, tissue was digested in a solution containing collagenase/dispase (0.227 mg/mL, Sigma-Aldrich) and DNase (50 U/ml, Roche) at 37°C for 45 minutes. For isolating astrocytes, tissue was instead triturated using a 10 mL pipette, then digested in a solution containing Papain (4 U/mL) (Worthington Biochemical, Cat#LS003126) at 37°C for 45 minutes, with repeated trituration every 15 minutes. Digested brains were then passed through a 70-μm strainer (Corning) and washed with cRPMI. Myelin was separated out from mononuclear cells by resuspending samples in 20 mL of 40% Percoll (Cytiva, Cat#17-0891-02) and centrifuging at 650g for 25 minutes. Myelin was aspirated, and the remaining cell pellets were washed in cRPMI, resuspended, and kept on ice until plating. For *ex vivo* cytokine stimulation, cells were resuspended in cRPMI with Brefeldin A (20 μg/ml) (Selleckchem, Cat#S7046) or Brefeldin A, Phorbol 12-Myristate 13-Acetate (PMA) (200 ng/ml) (Sigma Aldrich, Cat#P1585), and Ionomycin (1 μg/ml) (Sigma Aldrich, Cat#I0634) for 5 hours at 37° C.

Spleens were harvested into cold cRPMI, mechanically homogenized, and passed through a 40-μm strainer (Fisher Scientific, Cat#08-771-1). Cells were resuspended in red-blood cell (RBC) lysis buffer (0.16M NH_4_Cl) for 2 minutes. Samples were washed and resuspended with cRPMI and kept on ice until plating.

Cells counts were acquired by diluting 1:10 in 0.4% trypan blue solution (Sigma-Aldrich Cat#T8154) and counted on a hemocytometer (Hausser Scientific Cat#3110) using a DM 2000 LED brightfield microscope (Leica).

### Flow Cytometry:

Single cell suspensions were plated in a 96 well plate and subsequently resuspended in Fc Block, comprised of FACS buffer (1X PBS, 0.2% BSA, and 2mM EDTA) with 0.1 μg/mL 2.4G2 Ab (BioXCell, Cat#CUS-HB-197) and 0.1% rat gamma globulin (Jackson Immunoresearch, Cat#012-000-002) for 10 minutes. Cells were stained for surface markers and, for extracellular T cell and myeloid panels, eBioscience fixable live/dead viability dye 780 (1:800, Thermo Fisher Scientific, Cat#50-112-9035) or, for intracellular cytokine staining panel, eBioscience fixable live/dead viability dye efluor506 (1:800, Thermo Fisher Scientific, Cat#65-0866-14) for 30 minutes at 4°C. Cells were then washed twice with 50 μL FACS buffer. For intracellular staining, cells were fixed with fixation/permeabilization solution (eBioscience, 005123-43 and 00-5223-56) overnight at 4°C. Cells were then washed twice with 50 μL permeabilization buffer (eBioscience, 00-8333-56) and stained for intracellular markers in 1X perm buffer for 30 minutes at room temperature. Subsequently, they were washed twice with 1X perm buffer and finally resuspended in 200 μL FACS buffer. They were then acquired on a 3 or 5 laser Cytek Aurora Flow Cytometry System or the Gallios Flow Cytometer. Data was analyzed using FlowJo software v10.9.0.

The following antibodies at 1:200 were used: CD45-AF700 (BioLegend, Cat#103128), CD45-eFlour 450 (Thermo Scientific, Cat#48-0451-82) CD11b-PerCP Cy5.5 (Thermo Fisher Scientific, Cat#45-0112-80), iNOS-APC (Thermo Fisher Scientific, Cat#17-5920-82), MHCII-Super Bright 780 (Thermo Fisher Scientific, Cat#78-5321-82), CD4-BV650 (Thermo Fisher Scientific, Cat#563232), CD8-BV421 (Thermo Fisher Scientific, Cat#563898), CD8-PerCP-Cy5.5 (Thermo Fisher Scientific, Cat#45-0081-82 ), Foxp3-eFlour 450 (Thermo Fisher Scientific, Cat#48-5773-82), IFNγ-PerCPCy5.5 (Thermo Fisher Scientific, Cat#45-7311-82), TNFα-PE (Thermo Fisher Scientific, Cat#12-7321-81), TCRβ-APC (Thermo Fisher Scientific, Cat#17-5961-81), Ly6C-PE (Thermo Fisher Scientific, Cat#12-5932-82), Ly6C-PE/Cy7 (Thermo Fisher Scientific, Cat#25-5932-82), Ki67-PE/Cy7 (Thermo Fisher Scientific, Cat#25-5698-82), B220-PE-Cy5 (Thermo Fisher Scientific, Cat#15-0452-82), NK1.1-SB780 (Thermo Scientific, Cat#78-5941-82), Ly6g-BV711 (Biolegend, Cat#127643), H2K^b^/H2D^b^-PE/Cy7 (BioLegend, Cat#114616), H2K^b^/H2D^b^-PE (BioLegend, Cat# 114608). The following antibodies were used at 1:50 dilution: GLAST-PE (Miltenyi Biotech, Cat#130-118-344). The follow antibody was used at a 1:20 dilution: Granzyme-B-APC (Thermo Fisher Scientific, Cat#GRB05).

### Astrocyte Purification:

For purification of astrocytes, brains were harvested and processed as described above. ACSA2 + astrocytes were then isolated by magnetic bead enrichment according to manufacturer protocol (Miltenyi Biotech, Cat# 130-097-678).

### Immunofluorescence:

For immunofluorescence, brains were bisected along the sagittal midline and either immediately fresh frozen on dry ice or fixed in cold 4% PFA (EMS Cat#15710-S) for 24 hr at 4° C. Fixed brains were then cryoprotected in 30% sucrose for 24 hr at 4° C, embedded in OCT (Tissue Tek Cat#25608-930), and frozen on dry ice. Tissue blocks were stored at −20°C until needed for further analysis. 30–50 μm fixed sections were then prepared using a CM1950 cryostat (Leica) and stored in 1X PBS as free-floating sections. For fresh frozen tissue, 10–15 μm sections were immediately mounted onto charged glass slides (Fisher Scientific Cat#1255015) and allowed to dry at room temperature overnight prior to staining. To immunostain brain sections, the slices were first incubated in a blocking solution [2% normal donkey serum] (Jackson ImmunoResearch Cat#017-000-121), 1% BSA, 0.05% Tween 20 (Fisher Scientific Cat#BP337), and 0.5% Triton X-100 (Sigma-Aldrich Cat#028SK001) in 1 X PBS) at room temperature for 1 hour. Then, tissue was stained for 1 hour at room temperature or overnight at 4° C with primary antibodies in blocking solution. Samples were washed three times in 0.05% Tween 20 solution and stained with secondary antibodies for 1 hr at room temperature in blocking solution. Finally, tissues were washed three times and mounted onto glass slides using AquaMount (Polysciences Cat#18606), and coverslipped (Globe Scientific Cat#1419). In some experiments, the tissue was counter-stained with DAPI (ThermoScientific Cat#62248) and washed just before mounting onto slides. Slides were dried, AquaMount (Polysciences Cat#18606) was applied, and coverslipped (Globe Scientific Cat#1419).

Primary antibodies included: anti-Me49 (1:10,000 dilution) (gift from Fausto Araujo), GFAP (1:200 dilution) (DAKO Cat#Z0334) or (Invitrogen Cat#130300), MHCI (1:100 dilution) (Abcam Cat#ab15681). Secondary antibodies were used at 1:400 dilution. To stain Me49 and GFAP (rabbit): donkey anti-rabbit-AF594 (Jackson Cat#711585152); to stain MHCI and GFAP (rat): donkey anti-rat-AF647 (Jackson Cat#712605150). Images were acquired using a Leica Stellaris 5 confocal microscope and processed using Fiji software^86^.

### Statistical Analysis:

All data was graphed in GraphPad Prism 9. Statistical analyses were performed using Prism software (v8.4) or RStudio (v 4.4.2) statistical packages. A two-tailed Student’s t-test was used to compare two independent groups. To account for biological variation between experiments, data compiled from experimental replicates was analyzed in R using a randomized block ANOVA, where experimental groups were modeled as a fixed effect and experimental day as a random effect^87^. For time course experiment data, gene expression data were log_2_-transformed to improve normality, and differences across timepoints were evaluated using one-way ANOVA with Tukey’s post hoc multiple comparisons test. Outliers were identified and removed using ROUTs method with a Q value of 1^88^. Data from flow cytometric analyses and qPCR results were graphed using Graph-Pad Prism and data related to transcriptomic analyses were graphed using R. Error bars indicate standard error of the mean (s.e.m). The test used for each experiment is denoted in the figure legend, and *p*-values are denoted with ns = not significant, p < 0.05(*), p < 0.01(**), and p < 0.001 (***).

## Supplementary Material

This is a list of supplementary files associated with this preprint. Click to download.

- Supplementaldatacombinedfinal.docx

## Data Availability

All data needed to support the conclusions of this paper are present in the paper and/or the Supplementary Materials, with all data points shown. The dataset supporting the conclusions of this article will be available in FigShare upon publication. Bulk RNA-sequencing data will be available on GEO upon publication.

## Abbreviations

CNS: Central Nervous System
TE: Toxoplasmic encephalitis
MHCI: Major histocompatibility complex I
MHCII: Major histocompatibility complex II
DEGs: Differentially expressed genes
GO: Gene ontology
qPCR: Quantitative polymerase chain reaction
APC: Antigen presenting cell
iNOS: Inducible nitric oxide synthase
AIDS: Acquired Immunodeficiency Syndrome
BBB: Blood-brain barrier
IFNg: Interferon-gamma
cDC1s: Conventional type 1 dendritic cells
cDC2s: Conventional type 2 dendritic cells
EAE: Experimental autoimmune encephalitis
BEC: Brain endothelial cell
b2m: Beta-2 microglobulin
PBS: Phosphate buffered saline
RT-qPCR: Reverse transcription-quantitative polymerase chain reaction
Wpi: Weeks post infection
RNA: Ribonucleic acid
TNFa: Tumor necrosis factor alpha
DNA: Deoxyribonucleic acid
MFI: Mean fluorescence intensity
GFAP: Glial fibrillary acidic protein
GrzmB: Granzyme-B
TMEV: Theiler’s murine encephalomyelitis virus
HFFs: Human foreskin fibroblasts
BFA: Brefeldin A
PMA: Phorbol 12-Myristate 13-Acetate
FACS: Fluorescence-activated cell sorting
ANOVA: Analysis of Variance
